# The regulation of mitochondrial DNA copy number in glioblastoma cells

**DOI:** 10.1038/cdd.2013.115

**Published:** 2013-08-30

**Authors:** A Dickinson, K Y Yeung, J Donoghue, M J Baker, R DW Kelly, M McKenzie, T G Johns, J C St. John

**Affiliations:** 1 The Mitochondrial Genetics Group, Centre for Genetic Diseases, Monash Institute of Medical Research, Monash University, 27-31 Wright Street, Clayton, Victoria 3168, Australia; 2 Molecular Basis of Metabolic Disease, Division of Metabolic and Vascular Health, Warwick Medical School, The University of Warwick, Clifford Bridge Road, Coventry, CV2 2DX, UK; 3 The Oncogenic Signalling Laboratory, Centre for Cancer Research, Monash Institute of Medical Research, Monash University, 27-31 Wright Street, Clayton, Victoria 3168, Australia; 4 Department of Biochemistry and Molecular Biology, Bio21 Institute, University of Melbourne, Melbourne, Victoria, Australia; 5 The Molecular Basis of Mitochondrial Disease Group, Centre for Genetic Diseases, Monash Institute of Medical Research, Monash University, 27-31 Wright Street, Clayton, Victoria 3168, Australia

**Keywords:** mitochondrial DNA (mtDNA), glioblastoma multiforme (GBM), tumorigenesis, mtDNA copy number, mtDNA set point, ATP

## Abstract

As stem cells undergo differentiation, mitochondrial DNA (mtDNA) copy number is strictly regulated in order that specialized cells can generate appropriate levels of adenosine triphosphate (ATP) through oxidative phosphorylation (OXPHOS) to undertake their specific functions. It is not understood whether tumor-initiating cells regulate their mtDNA in a similar manner or whether mtDNA is essential for tumorigenesis. We show that human neural stem cells (hNSCs) increased their mtDNA content during differentiation in a process that was mediated by a synergistic relationship between the nuclear and mitochondrial genomes and results in increased respiratory capacity. Differentiating multipotent glioblastoma cells failed to match the expansion in mtDNA copy number, patterns of gene expression and increased respiratory capacity observed in hNSCs. Partial depletion of glioblastoma cell mtDNA rescued mtDNA replication events and enhanced cell differentiation. However, prolonged depletion resulted in impaired mtDNA replication, reduced proliferation and induced the expression of early developmental and pro-survival markers including *POU class 5 homeobox 1* (*OCT4*) and *sonic hedgehog* (*SHH*). The transfer of glioblastoma cells depleted to varying degrees of their mtDNA content into immunocompromised mice resulted in tumors requiring significantly longer to form compared with non-depleted cells. The number of tumors formed and the time to tumor formation was relative to the degree of mtDNA depletion. The tumors derived from mtDNA depleted glioblastoma cells recovered their mtDNA copy number as part of the tumor formation process. These outcomes demonstrate the importance of mtDNA to the initiation and maintenance of tumorigenesis in glioblastoma multiforme.

The circular, double-stranded human mitochondrial genome (mitochondrial DNA, mtDNA) is 16 569 bp in size and encodes 13 subunits of the electron transfer chain (ETC),^1^ which is the major generator of cellular adenosine triphosphate (ATP) through oxidative phosphorylation (OXPHOS).^2^ It also possesses 22 transfer RNAs and 2 ribosomal RNAs, and one non-coding region, the D-loop,^1^ which is the site of interaction for the nuclear-encoded mtDNA replication factors.^3^ MtDNA replication is initiated by mitochondrial transcription factor A (TFAM),^4^ which generates the primer used by the catalytic subunit of the mtDNA-specific DNA polymerase, polymerase *γ* A (POLGA), to copy mtDNA. Replication is supported by POLGA's accessory subunit, polymerase *γ* B (POLGB), the mtDNA-specific helicase, TWINKLE, and the mtDNA single-stranded-binding protein (MTSSB).^5, 6^

Regulation of mtDNA copy number is essential for maintaining cellular energy requirements. High-energy requiring cells, such as muscle and neurons, require large quantities of ATP and maintain high numbers of mtDNA copy while low-energy requiring cells, spleen and endothelial cells, maintain fewer copies.^7^ MtDNA replication and transcription are tightly coupled such that the expression of the mtDNA genes, and hence the generation of ATP through OXPHOS, requires continuous replication of mtDNA.^8^

Metabolism in tumor-initiating cells is described by the Warburg effect. Tumors utilize aerobic glycolysis even under normoxic conditions, which normally promotes OXPHOS.^9^ This promotes self-renewal and the highly proliferative nature of tumor cells,^10^ enabling them to generate sufficient energy and pools of metabolic intermediates. This is similar to embryonic stem cells (ESCs), which are highly proliferative, undergo self-renewal^11^ and maintain few copies of mtDNA.^12^ This establishes the mtDNA set point, whereby ESCs accumulate mtDNA in a cell-specific manner to meet the functional requirements of specialized cells during differentiation.^12, 13^ This requires synchrony between the nuclear and mtDNA genomes to ensure that mtDNA replication and differentiation take place concurrently.

It is not known whether precursor cells giving rise to tumors can mimic the mtDNA replication events of stem cells as they differentiate into somatic cells and whether they require mtDNA to promote tumorigenesis. Glioblastoma multiforme (GBM) is the most common subgroup of highly malignant astrocytomas with a median survival time of 12 to 16 months.^14^ Cell lines derived from GBM tumors, which are dependent on aerobic glycolysis,^15, 16^ are excellent models to understand the role that mtDNA has in tumor formation and self-renewal. The best characterized of the GBM cell lines is HSR-GBM1. It expresses neural stem cell (NSC) makers, such as NESTIN, MUSASHI1 and prominin 1 (CD133) and their levels of expression correlate with patient prognosis.^17^ They differentiate into neurons and astrocytes and express the astrocyte end point marker, glial fibrillary acidic protein (GFAP).^18^

We have determined whether GBM cells can modulate mtDNA copy number and chromosomal gene expression during differentiation when compared with human (h)NSCs. Furthermore, we have determined whether mtDNA is essential for the differentiation of GBM cells, their survival and the initiation of tumorigenesis by depleting them of mtDNA and allowing them to recover *in vitro* and *in vivo*. We demonstrate that mtDNA is essential to tumorigenesis.

## Results

### GBM cells do not expand mtDNA copy number during differentiation

**Figure 1:**
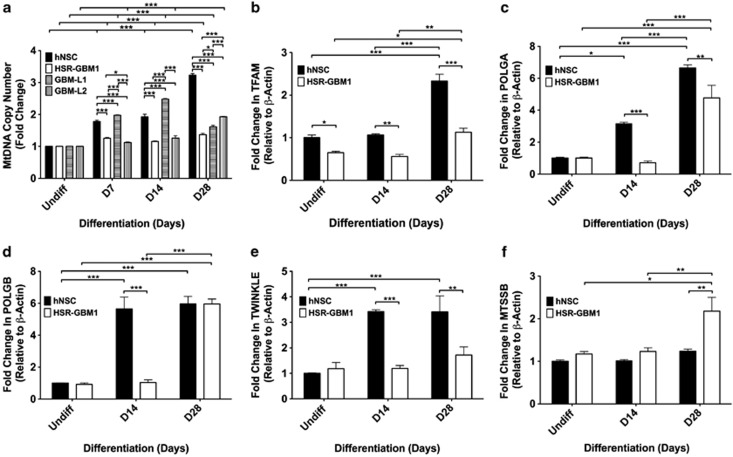
MtDNA copy number and gene expression in differentiating GBM cells and hNSCs. Mean mtDNA copy number in differentiating hNSCs, HSR-GBM1, GBM-L1 and GBM-L2 cells (**a**). Gene expression analysis of the nuclear-encoded mtDNA replication and transcription factors in differentiating HSR-GBM1 cells and hNSCs (**b–f**). Fold change in expression relative to undifferentiated cells, weighted to *β-ACTIN*, for *TFAM* (**b**), *POLGA* (**c**), *POLGB* (**d**), *TWINKLE* (**e**) and *MTSSB* (**f**). Bars represent mean values±S.E.M. *P<0.05, **P<0.01 and ***P<0.001

To determine whether GBM cells can modulate mtDNA copy number during differentiation, we induced three human GBM (HSR-GBM1, GBM-L1 and L2) and one human neural stem cell (hNSC) lines to undergo astrocyte differentiation (Figure 1a). hNSCs increased mtDNA copy number progressively resulting in a 3.23-fold increase by day 28 (*P*<0.001). Although HSR-GBM1 cells significantly increased (1.25-fold) mtDNA copy number on day 7 (*P*<0.001), this only increased to 1.37-fold by day 28 (*P*<0.001). A similar pattern was observed for GBM-L2 cells, except they had significantly more copies than HSR-GBM1 and GBM-L1 cells on day 28 (*P*<0.001). Although GBM-L1 cells displayed significant increases on days 7 and 14, levels were reduced by day 28. Nevertheless, cells from all three GBM lines had significantly fewer copies of mtDNA than the hNSCs by day 28 (*P*<0.001).

### GBM cells do not maintain elevated levels of GFAP expression during differentiation

We determined whether GBM cells concordantly regulate the expression of NSC and astrocyte-specific genes during differentiation. Although hNSCs maintained expression of *NESTIN*, *MUSASHI1* and *CD133* throughout differentiation, *GFAP* expression significantly increased at each time point. Although HSR-GBM1 cells reduced expression of multipotent genes by 28 days of differentiation (Supplementary Figures S1A–C), they did not sustain increased *GFAP* expression (Supplementary Figure S1D). For GBM-L1 cells, expression increased progressively for all four genes (Supplementary Figures S2A–D), while GBM-L2 cells only increased expression up to day 14, which decreased thereafter (Supplementary Figures S2E–H).

### GBM cells do not upregulate expression of the nuclear-encoded mtDNA replication factors during differentiation

As GBM cells fail to expand mtDNA copy number during astrocyte-induced differentiation, we analyzed the expression of the nuclear-encoded mtDNA replication factors in HSR-GBM1 cells and compared them with hNSCs. During hNSC differentiation, *TFAM* (Figure 1b), *POLGA* (Figure 1c), *POLGB* (Figure 1d), *TWINKLE* (Figure 1e) and *MTSSB* (Figure 1f) progressively increased or maintained expression to match increases in mtDNA copy number. Patterns of gene expression for HSR-GBM1 cells were dissimilar to hNSCs. Although levels of *POLGA* (Figure 1c) expression increased on day 28 in HSR-GBM1 cells, which were lower than for hNSCs (*P*<0.01), only *POLGB* (Figure 1d) had similar levels of expression to hNSCs on day 28 but not before this. Although expression of *MTSSB* in HSR-GBM1 cells was similar to hNSCs on day 14, it was significantly higher than for hNSCs on day 28 (Figure 1f). Consequently, GBM cells do not concordantly regulate expression of the nuclear-encoded mtDNA replication factors and markers of early and late differentiation, resulting in their failure to expand mtDNA copy number.

### GBM cells do not increase their respiratory capacity during differentiation

To determine whether the failure of GBM cells to increase mtDNA copy number during differentiation altered oxygen (O_2_) consumption rates, ATP content and lactate production, we compared HSR-GBM1 cells and hNSCs (Supplementary Table SI). Undifferentiated HSR-GBM1 cells consumed significantly more O_2_ than undifferentiated hNSCs (*P*<0.001). Uncoupling of the ETC revealed that undifferentiated hNSCs and HSR-GBM1 cells were respiring at maximal rate based on limited reserve capacities of 0.9 and 1.1, respectively. During differentiation, hNSCs increased O_2_ consumption rates by 2.46-fold (*P*<0.001) and ETC reserve capacity (*P*<0.001). However, HSR-GBM1 cells marginally increased O_2_ consumption rates and ETC reserve capacity. The increased respiratory capacity of differentiated hNSCs represented a fourfold increase in ATP content relative to undifferentiated hNSCs (*P*<0.001), which was less profound in HSR-GBM1 (*P*<0.001). Undifferentiated HSR-GBM1 cells secreted significantly more lactate than hNSCs (*P*<0.001), which was reduced following differentiation (*P*<0.001). Consequently, increased O_2_ consumption and OXPHOS-generated ATP are dependent on increased mtDNA copy number.

### MtDNA depletion of HSR-GBM1 cells

**Figure 2:**
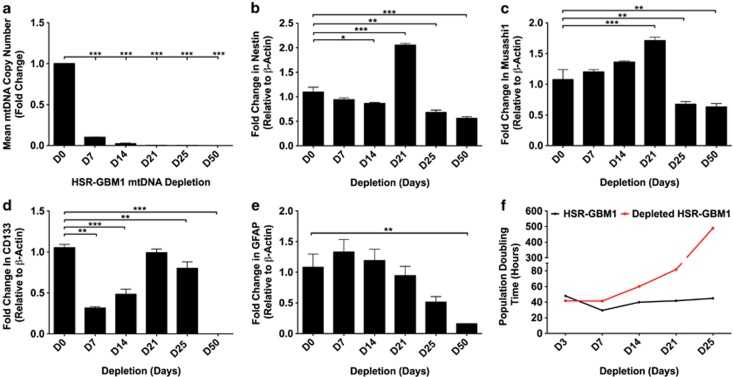
MtDNA depletion of GBM cells. Mean mtDNA copy number was assessed over 50 days of mtDNA depletion (**a**). Gene expression analysis of undifferentiated and mtDNA depleted HSR-GBM1 cells was determined relative to *β*-ACTIN and as fold changes relative to non-depleted cells for *NESTIN* (**b**), *MUSASHI1* (**c**), *CD133* (**d**) and *GFAP* (**e**). Population-doubling times of undifferentiated and mtDNA depleted HSR-GBM1 cells (**f**). Columns represent mean values±S.E.M. *P<0.05, **P<0.01 and ***P<0.001

We determined whether mtDNA copy number was essential for tumor cell survival. HSR-GBM1 cells were depleted of their mtDNA using 10 *μ*M 2'-3'-dideoxycytidine (ddC).^19^ Over 50 days, copy number progressively decreased (*P*<0.001) to <1 by day 25 onward (Figure 2a).

We then determined whether mtDNA depletion induced changes in expression of *NESTIN*, *MUSASHI1*, *CD133* and *GFAP*. There were differential patterns of expression for *NESTIN* (Figure 2b) and *MUSASHI1* (Figure 2c) after 7 and 14 days of depletion. However, expression of both genes was upregulated by day 21 and downregulated by days 25 and 50. *CD133* expression was significantly reduced after 7 and 14 days (*P*<0.001; Figure 2d), returned to basal levels by day 21, and was undetectable by day 50 (*P*<0.001). There were no significant changes in *GFAP* expression during the first 14 days. However, from day 21 onward, expression progressively decreased (Figure 2e).

As there were significant decreases in multipotent gene and *GFAP* expression during mtDNA depletion, we analyzed expression profiles of non-depleted and cells depleted for 25 and 50 days using a neural real-time PCR array (see Supplementary Table SII). Days 25 and 50 depleted cells showed significant and differential expression of 26/80 genes relative to non-depleted cells. Genes upregulated in both depleted groups are associated with growth factor signaling, anti-apoptosis and cell adhesion, namely fibroblast growth factor 13 (*FGF13*), glial-derived neurotrophic factor (*GDNF*) and semaphorin-4D (*SEMA4D*). The cell pro-proliferation factor, vascular endothelial growth factor A (*VEGFA*), was upregulated on day 25 and the anti-proliferation factor, *anaplastic lymphoma kinase*, was upregulated by day 50. By day 50, proliferation rates for depleted cells were reduced (Figure 2f). These outcomes suggest that HSR-GBM1 cells require sufficient copies of mtDNA to support cell proliferation.

The early neural patterning factor, sonic hedgehog (*SHH*), was upregulated in day 50 depleted cells while levels of expression for acetylcholinesterase (*ACHE*), dopamine receptor D2 (*DRD2*) and neuronal pentraxin 1 (*NPTX*) were upregulated by day 25 but decreased by day 50. Similarly, the regulators of cell fate and differentiation, *apolipoprotein E* (*APOE*), *Achaete-scute homolog 1* (*ASCL1*) and *delta-like 1* (*DLL1*) were downregulated while *hairy/enhancer-of-split related with YRPW motif* (*HEY1*), a transcriptional repressor involved in neurogenesis, was upregulated in days 25 and 50 depleted cells. Consequently, mtDNA depletion leads to increased expression of genes associated with early developmental processes.

**Figure 3:**
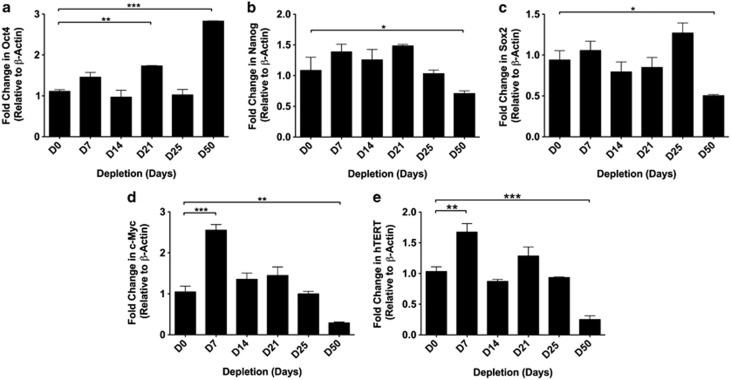
Gene expression analysis of markers of pluripotency and self-renewal in undifferentiated and mtDNA depleted GBM cells. Gene expression for undifferentiated and mtDNA depleted HSR-GBM1 cells was determined relative to *β-ACTIN* and as fold changes relative to non-depleted cells for *OCT4* (**a**), *NANOG* (**b**), *SOX2* (**c**), *c-MYC* (**d**) and *hTERT* (**e**). Bars represent mean values±S.E.M. *P<0.05, **P<0.01 and ***P<0.001

As there were significant increases in expression of early developmental markers, we analyzed *POU class 5 homeobox 1* (*OCT4*), *Nanog homeobox* (*NANOG*), *sex determining region Y-box 2* (*SOX2*), *V-Myc myelocytomatosis viral oncogene homolog* (*Avian*) (*c-MYC*) and *human telomerase reverse transcriptase* (*hTERT*), which are associated with pluripotency, cell proliferation and self-renewal. Although the expression of *OCT4* (Figure 3a), *NANOG* (Figure 3b), *SOX2* (Figure 3c), *c-MYC* (Figure 3d) and *hTERT* (Figure 3e) fluctuated over the first 25 days of mtDNA depletion, only *OCT4* expression increased (threefold) by day 50 (*P*<0.001).

### Replenishment of mtDNA copy number following prolonged mtDNA depletion

**Figure 4:**
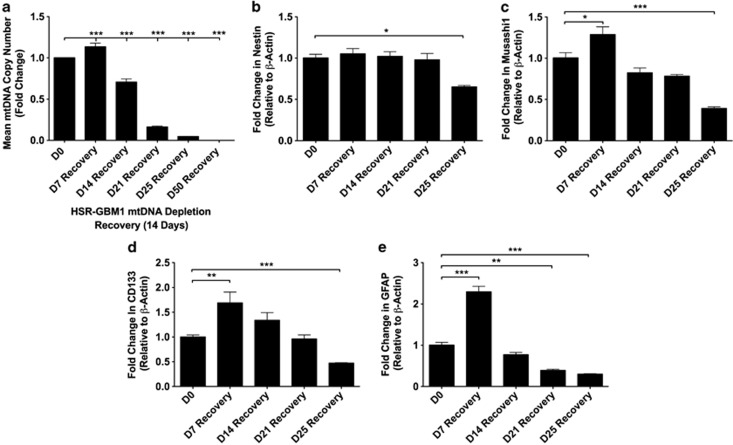
Recovery of mtDNA copy number and gene expression in depleted GBM cells. Mean (±S.E.M.) mtDNA copy number following 14 days of recovery for undifferentiated HSR-GBM1 cells depleted for up to 50 days (**a**). Gene expression relative to *β-ACTIN* for HSR-GBM1 cells following 14 days of recovery after depletion for up to 25 days. Fold change in expression of *NESTIN* (**b**), *MUSASHI1* (**c**), *CD133* (**d**) and *GFAP* (**e**) is relative to non-depleted GBM cells. Bars represent mean values±S.E.M. *P<0.05, **P<0.01 and ***P<0.001

To determine whether replenishment of mtDNA copy number influenced gene expression of undifferentiated HSR-GBM1 cells, we analyzed cells depleted of mtDNA for 7, 14, 21, 25 and 50 days followed by recovery for 14 days (Figure 4a). HSR-GBM1 cells recovering after 7 days of depletion re-established copy number to levels 1.12 higher than non-depleted cells (*P*<0.05), demonstrating that mtDNA depletion is reversible. However, cells recovering from 14, 21 and 25 days failed to fully replenish copy number with levels 1.40-, 6.60- and 23.30-fold lower than for non-depleted cells, respectively (*P*<0.001). Cells depleted for 50 days failed to replenish mtDNA. Consequently, depletion as far as 7 to 14 days enables cells to fully replenish mtDNA. However, recovery of cells in conditioned media from non-depleted cells enabled cells depleted for 14 and 21 days to replenish copy number to levels significantly above or just below non-depleted cells, respectively (Supplementary Figure S3A).

As mtDNA replenishment was compromised following prolonged depletion in non-conditioned media, we analyzed expression of *NESTIN*, *MUSASHI1*, *CD133* and *GFAP* during 14 days of recovery. *NESTIN* remained unaffected in cells recovering from 7, 14 and 21 days of mtDNA depletion (Figure 4b). For *MUSASHI1* (Figure 4c), *CD133* (Figure 4d) and *GFAP* (Figure 4e), there were significant increases in cells recovering from 7 days depletion. However, there were decreases for all four genes in cells recovering from 25 days of depletion. Recovery in conditioned media resulted in down regulation of *NESTIN*, *MUSASHI1* and *GFAP* expression (Supplementary Figures S3B–E). These outcomes further emphasize the asynchronous relationship between the nuclear and mitochondrial genomes in HSR-GBM1 cells. Furthermore, depletion had no adverse effects on optic atrophy 1 (OPA1) processing indicating that mitochondrial networks were not disrupted through microtubule-associated protein 1 light chain 3 beta (LC3B), though autophagy was upregulated during depletion and reduced during recovery (Supplementary Figure S3F).

**Figure 5:**
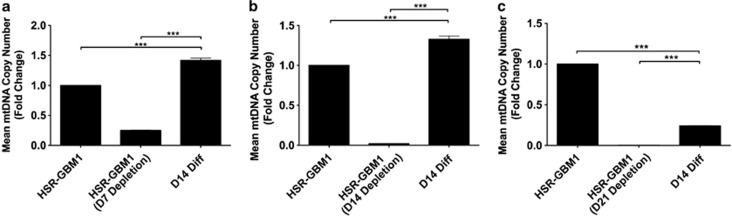
MtDNA copy number in mtDNA depleted, differentiating GBM cells. MtDNA copy for HSR-GBM1 cells depleted for 7 days (**a**), 14 days (**b**) and 21 days (**c**) and differentiated for 14 days are shown as fold changes relative to non-depleted HSR-GBM1 cells. Columns represent mean values±S.E.M. ***P<0.001

To determine whether modulation of mtDNA copy number promotes cellular differentiation, we induced HSR-GBM1 cells depleted for 7, 14 and 21 days to differentiate into astrocytes for 14 days. Day 7 depleted cells replenished copy number by day 14 of differentiation to levels 1.46-fold higher than observed in day 0 cells (*P*<0.001; Figure 5a). Similarly, day 14 depleted cells reached levels 1.32-fold greater by day 14 of differentiation (Figure 5b). However, surviving cells depleted for 21 days increased copy number to >4-fold lower than day 0 (*P*<0.001; Figure 5c).

Following 7 days of depletion and 14 days of differentiation, expression of *NESTIN* (Supplementary Figure S4A) and *CD133* (Supplementary Figure S4C) was unchanged, whereas *MUSASHI1* was elevated (*P*<0.05; Supplementary Figure S4B) and *GFAP* was significantly upregulated (*P*<0.001; Supplementary Figure S4D). Following differentiation of cells depleted for 14 days, there were significant reductions in expression for *NESTIN* (*P*<0.001; Supplementary Figure S4E) and *CD133* (*P*<0.001; Supplementary Figure S4G) compared with depleted cells but not for *MUSASHI1* (*P*>0.05; Supplementary Figure S4F), whereas *GFAP* was significantly increased (*P*<0.001; Supplementary Figure S4H). HSR-GBM1 cells depleted for 21 days and induced to differentiate exhibited a significant reduction in *NESTIN* (3.91-fold; *P*<0.001; Supplementary Figure S4J), *MUSASHI1* (2.44-fold; *P*<0.001; Supplementary Figure S4K) and *CD133* expression (33.00-fold; *P*<0.001; Supplementary Figure S4L) compared with depleted cells. However, *GFAP* expression was significantly upregulated (12.54-fold; *P*<0.001; Supplementary Figure S4M). These outcomes suggest that a population of HSR-GBM1 cells can initiate astrocyte differentiation in the presence of reduced mtDNA copy number.

### Recovery of mtDNA copy number in tumor-forming mtDNA depleted HSR-GBM1 cells

**Figure 6:**
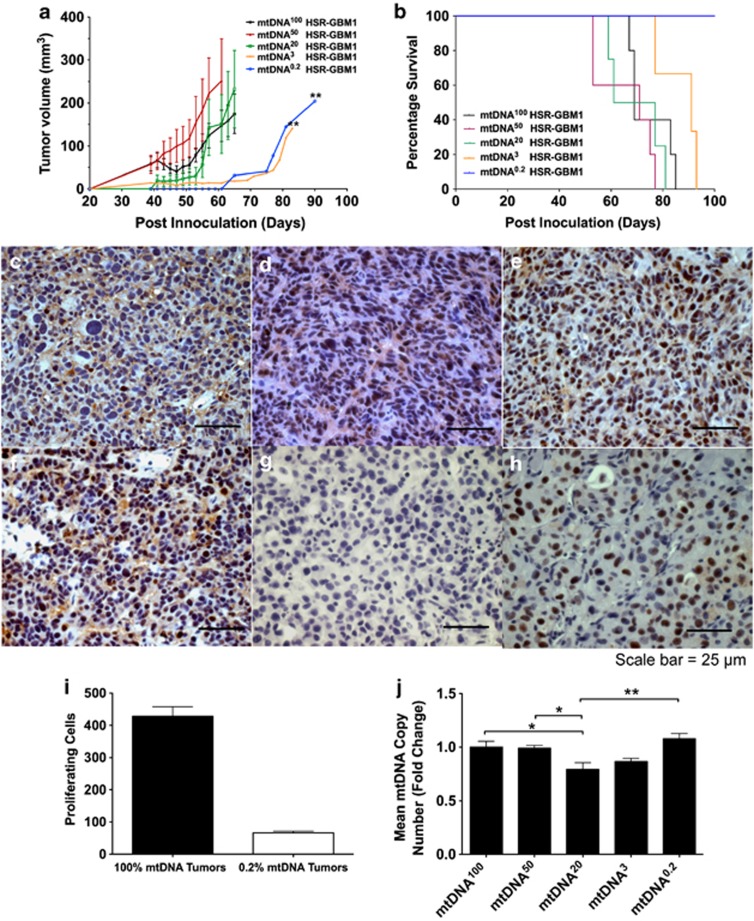
HSR-GBM1 tumor formation assay and assessment of mtDNA copy number. Tumor growth curve analysis of mtDNA depleted (mtDNA^50^, mtDNA^20^, mtDNA^3^ and mtDNA^0.2^) and non-depleted (mtDNA^100^) HSR-GBM1 cells (**a**). Kaplan–Meier survival plot for non-depleted and depleted HSR-GBM1 cells (**b**). Immunohistochemical labeling of proliferating cell nuclear antigen. in depleted (**c**) and non-depleted (**d–f**) HSR-GBM1 tumors. Negative control (**g**) and positive control (**h**). Quantification of proliferating cell nuclear antigen positive cells in depleted and non-depleted HSR-GBM1 tumors (**i**). Mean mtDNA copy number analysis of depleted and non-depleted HSR-GBM1 tumors (**j**). Columns represent mean values±S.E.M. *P<0.05 and **P<0.01. Scale bars=25 *μ*m

We then assessed the tumorigenic potential of cells depleted to varying levels. We depleted HSR-GBM1 cells to ∼50% (mtDNA^50^), 20% (mtDNA^20^), 3% (mtDNA^3^) and 0.2% (mtDNA^0.2^) of their original mtDNA content. These and non-depleted (mtDNA^100^) cells were transferred into Bagg albino (Balb/c) nude mice. During the first 40 days post-inoculation, tumors from the mtDNA^100^ and mtDNA^50^ cells developed at a faster rate than mtDNA^20^, mtDNA^3^ and mtDNA^0.2^ cells (Figure 6a) although this was not statistically significant. After 40 days, tumors from mtDNA^50^ cells grew at an accelerated rate compared with mtDNA^100^ tumors and this trend was maintained. Tumors from mtDNA^20^ cells developed slowly until day 55 and, by day 65, developed faster than mtDNA^100^ tumors. Tumors derived from mtDNA^3^ and mtDNA^0.2^ cells showed delayed development compared with mtDNA^100^ tumors (*P*<0.01). The frequency of tumor formation was inversely related to mtDNA depletion. MtDNA^100^ cells generated 11/12 tumors of which 1 regressed; 10/12 were derived from mtDNA^50^ cells and 2 regressed; 6/12 were generated from mtDNA^20^ cells, 6/12 were derived from mtDNA^3^ cells and 3 regressed and 2/12 were generated from mtDNA^0.2^ cells. Tumor formation to 500 mm^3^ was least in the mtDNA^0.2^ and greatest in the mtDNA^100^ cohorts (Figure 6b). These data demonstrate that increased mtDNA depletion reduces the frequency of tumor formation.

To determine whether tumor formation was dependent on the recovery of mtDNA in mtDNA depleted cells, we analyzed the proliferative potential of the mtDNA non-depleted and depleted tumors. Tumors derived from mtDNA^0.2^ cells (Figures 6c and j) exhibited significantly lower proliferative potential compared with mtDNA^100^ counterparts (Figures 6d–f and j). We analyzed mtDNA copy number in all tumors using human-specific primers. All tumors derived from depleted cells restored mtDNA copy number during tumorigenesis to levels at or near to *in vitro* grown HSR-GBM1 cells. Although there was no difference in mtDNA copy number between mtDNA^100^, mtDNA^50^, mtDNA^3^ and mtDNA^0.2^ tumors, there was significantly lower copy number in mtDNA^20^ tumors (*P*<0.05; Figure 6k).

## Discussion

We have compared GBM cells with hNSCs. We selected ESC-derived hNSCs as they express NSC markers, such as *NESTIN, MUSASHI1, CD133* and those listed in Supplementary Table SII. Furthermore, their regulation of mtDNA copy number is similar to primary murine NSCs.^20^ We observed differential expression of the multipotent genes, *NESTIN, MUSASHI1* and *CD133* between hNSC and GBM cells during differentiation. However, HSR-GBM1 cells could not maintain elevated levels of *GFAP* expression at later stages of differentiation. Furthermore, HSR-GBM1 cells failed to expand mtDNA copy number during differentiation, which was reflected in altered patterns of expression for the nuclear-encoded mtDNA replication factors. GBM-L2 cells also failed to increase mtDNA copy number and *GFAP* expression, whereas GBM-L1 cells increased *GFAP* expression but failed to expand mtDNA copy number synchronously.

Since the generation of ATP through OXPHOS is coupled to the continuous replication of mtDNA,^8^ GBM cells do not have the capacity to utilize OXPHOS effectively, probably due to mtDNA mutations, which have been identified in GBM patient tumors.^21^ Tumor cell metabolism is normally defined by glycolysis, with increased glucose uptake and lactate production.^9^ Indeed, undifferentiated HSR-GBM1 cells failed to increase O_2_ consumption rates and copy number and had high levels of lactate production, which were downregulated during differentiation, unlike their hNSC counterparts. An enhanced glycolytic state provides multiple benefits to tumor cells including sufficient ATP and biosynthetic intermediates to support cell division and growth^15, 16, 22^ and the generation of NADPH, which is involved in redox control.^23^

Similarly, ESCs are rapidly proliferating cells that promote self-renewal and pluripotency by maintaining low mtDNA copy number and primarily use glycolysis to produce ATP.^12, 13, 24, 25, 26^ This establishes the mtDNA set point,^12, 13^ which ensures that, during differentiation, heart, neural and muscle cells acquire high numbers of mtDNA copy to utilize OXPHOS,^7^ whereas endothelial cells possess few copies and rely on glycolysis.^27^

GBM is derived from multiple origins, such as glial cells^28^ and/or NSCs^29^ that have undergone neoplastic transformation. The abnormal regulation of mtDNA copy number in HSR-GBM1 cells is possibly indicative of a transformed glial cell. During transformation, the acquisition of aberrant oncogenic signaling^30^ and reactivation of pluripotent and multipotent regulators, such as OCT4, NANOG,^31^ SOX2,^32^ c-MYC^33^ and NESTIN,^17^ likely leads to abnormal regulation of mtDNA copy number. Pluripotent stem cells can be derived by reprogramming somatic cells through the forced expression of pluripotent factors.^34^ However, these cells often fail to re-establish the mtDNA set point and do not accumulate sufficient numbers of tDNA copy during differentiation.^25^ It appears that HSR-GBM1 cells regulate mtDNA in a similar manner to poorly induced pluripotent stem cells.

Following depletion for 7 days, HSR-GBM1 cells recovered and increased mtDNA copy number to levels higher than non-depleted cells. Furthermore, they exhibited increased expression of NSC markers and *GFAP*. This is similar to human neural progenitors, which survive short-term mtDNA depletion without losing multipotency.^35^ This also demonstrates that the short-term modulation of mtDNA copy number alters multipotent gene expression and triggers differentiation. However, days 7 to 14 of depletion represent the dividing line for HSR-GBM1 cells to restore mtDNA copy number whereby day 14 depleted cells do not completely restore mtDNA copy number while day 7 depleted cells increase mtDNA copy number in a similar manner to hNSCs. Nevertheless, cells recovered in conditioned media rescued mtDNA copy number but gene expression profiles were aberrantly regulated suggesting that other gene expression pathways were likely enacted.

The tumorigenicity of tumor cells depleted of mtDNA has been an unresolved issue with reports of increased^36^ and decreased^37^ tumorigenic potential in multiple tumor cell lines. The depletion of HSR-GBM1 cells to ∼90% (14 days of depletion) of their original mtDNA copy number did not reflect the significant changes in gene expression observed by day 50. By day 50, population-doubling times were increased, which could be explained by mtDNA depletion disrupting the synthesis of deoxyribose nucleoside triphosphates by mitochondria.^38^ We observed extensive changes in gene expression with reductions in *NESTIN*, *MUSASHI1* and *GFAP*, whereas *CD133*, which is a marker of bioenergetic stress during the very early stages of mtDNA depletion,^39^ was lost.

We depleted GBM cells of their mtDNA with ddC, which, at low concentrations, acts by directly inhibiting POLGA.^40^ At higher concentrations, it has been associated with cellular toxicity inducing neuropathies, liver disorders and lactic acidosis.^41^ Chemically induced mtDNA depletion has been associated with increased production of reactive O_2_ species, changes to mitochondrial ultrastructure and mitochondrial networks,^42, 43^ which may alter chromosomal gene expression.^42^ Nevertheless, our results indicate that mitochondrial stress responses were not activated, as demonstrated by L-OPA1 stability, which also suggests that cristae structures were not compromised. Furthermore, the unwanted organelle material would be degraded through the lysosomal machinery during depletion because of increased expression of LC3B. It is, therefore, likely that the changes in mtDNA levels are responsible for altered gene expression patterns.

Extended depletion of mtDNA did not induce cell death in HSR-GBM1 cells, an outcome that is likely to be driven by disruption of the mitochondrial apoptotic pathway caused by depletion of mtDNA^36^ and the increased expression we observed in anti-apoptotic genes. MtDNA depleted tumor cells have been reported to increase their anchorage-independent growth properties.^44^ We observed that HSR-GBM1 cells grew as tightly packed neurospheres and showed increased expression of cell adhesion-associated genes. Although GBM tumors have exhibited altered patterns of expression for the key regulators of pluripotency, *OCT4*, *NANOG*^31^ and *SOX2*,^32^ and *SHH* signaling,^45^ depleted HSR-GBM1 cells only exhibited elevated levels of *OCT4* and *SHH*.

Depleted HSR-GBM1 cells formed tumors in immunocompromised mice. However, the frequency was significantly hindered by mtDNA depletion. For those tumors that formed, mtDNA copy number recovered levels similar to *in vitro* cultured HSR-GBM1 cells demonstrating the necessity for tumor-initiating cells to establish a mtDNA set point. This is best exemplified by the mtDNA^0.2^ cells that possessed<1 copy of mtDNA per cell.

### Conclusion

We have established clear differences in the modulation of mtDNA copy number during differentiation between multipotent GBM cells and hNSCs. During differentiation, GBM cells failed to expand their mtDNA copy number and increase their respiratory capacity, which is underpinned by uncoordinated expression of nuclear-encoded mtDNA replication factors and lineage-specific markers. It is likely that a sub-population of cells exist with very few copies of mtDNA, which represents a tumor-sustaining population that, *in vivo*, has the capacity to re-establish its mtDNA set point after a prolonged period and generate tumors. This clearly demonstrates the importance of mtDNA to the establishment and maintenance of tumorigenesis.

## Materials and Methods

### Cell culture

HSR-GBM-1,^46^ GBM-L1 and GBM-L2 cells and hNSCs derived from the NIH-approved H9 (WA09) human ESC line (Invitrogen, Carlsbad, CA, USA) were cultured in complete neural stem cell media consisting of Dulbecco's modified essential medium/F12 (1 : 1), 2% StemPro neural supplement, 2 mM glutamax (all Gibco, Carlsbad, CA, USA), 20 ng/ml basic fibroblast growth factor and 20 ng/ml epidermal growth factor (both Millipore, Billerica, MA, USA) at 37 °C, 5% CO_2_. For mtDNA depletion, HSR-GBM1 cells were additionally cultured with 10 *μ*m ddC and 50 mg/ml uridine (both Sigma, St. Louis, MO, USA). For conditioned media experiments, media was collected from non-depleted cells each day and transferred to depleted cells after each media change.

### Differentiation assays

Undifferentiated GBM cells and hNSCs were seeded at a density of 2.5 × 10^4^ cells/cm^2^ in 20 mg/ml fibronectin coated six-well plates. They were cultured at 37 °C, 5% CO_2_ in astrocyte induction media consisting of Dulbecco's modified essential medium, 2 mM glutamax, 1% N2 supplement, 2% fetal bovine serum and 0.1 mM *β*-mecaptoethanol (all from Gibco).

### MtDNA copy number

Total DNA was extracted from cell samples using the DNeasy Blood and Tissue kit (Qiagen, Valencia, CA, USA) with proteinase K and RNase treatment, according to the manufacturer's instructions. To quantify mtDNA copy number, real-time PCR was performed using a 72-well Rotorgene-3000 (Corbett Research, Cambridge, UK) against external standards for mtDNA and *β*-globin, as previously described^47^ using primers listed in Supplementary Table SIII.

### Gene expression analysis

Total RNA was extracted using the RNeasy Mini Kit (Qiagen) with a DNase treatment step, according to the manufacturer's instructions. cDNA was synthesized using the Bioscript system (Bioline, London, UK) and PCR products were generated using RT-PCR and analyzed, as described in Facucho-Oliveira *et al.*^12^ Purified PCR products were prepared for each primer pair and used as standards in a series of 10-fold dilutions of known concentration for each gene to determine real-time PCR reaction efficiencies. For gene expression analysis, real-time PCR reactions contained 20 ng of total cDNA and primer sequences are described in Supplementary Table SIII. Data acquisition and melt curve analyses were performed, as described previously.^12^ Reactions were performed in triplicate twice. Relative gene expression was calculated using the Pfaffl method (mean±S.E.M.) and *β*-actin was selected as the housekeeping gene.^12^

### Cellular respiration analysis

O_2_ consumption rates for hNSCs and GBM cells were determined by high-resolution respirometry (Oroboros Oxygraph-2K, Innsbruck, Austria). Briefly, cells were dissociated using Accutase and cell numbers determined. Cells were resuspended in 50 *μ*l Hanks balanced salt solution and transferred into chambers containing 2 ml Hanks balanced salt solution maintained at 37 °C and continuously stirred at 750 r.p.m. O_2_ consumption was measured using the integrated software package Datlab (Version 3.1; Oroboros, Innsbruck, Austria), which presented respiration as O_2_ flux, pmol O_2_ per 10^6^ cells per second. Initial resting measurements (‘basal') were recorded for 5 min following O_2_ flux stabilization, after which a series of respiratory chain inhibitors were added at 10-min intervals to manipulate cellular respiration. In all, 5 mg/ml of the ATP synthase inhibitor oligomycin (Sigma) was used to assess mitochondrial coupling and the amount of non-phosphorylating respiration (‘non-phos'). Maximal uncoupled ETC respiratory capacity (‘uncoupled') was measured using 50–100 nM carbonyl cyanide p-(trifluoromethoxy) phenylhydrazone (FCCP; Sigma). In total, 5 mM of the complex III inhibitor Antimycin A (Sigma) was used to determine background O_2_ consumption and was subtracted from all calculated values.

### Total ATP and cellular lactate measurements

Cellular ATP content was determined using the ATPlite Luminescence ATP Detection Assay (PerkinElmer Life Sciences, Zaventem, Belgium), according to the manufacturer's instructions. Cellular lactate production was determined using the Lactate Assay Kit II (Biovision, San Francisco, CA, USA), according to the manufacturer's instructions. Cells were cultured under routine conditions and, before analysis, culture media completely removed and replaced with fresh media. After 24 h, a sample of media was removed for analysis. Luminescence (ATP) and absorbance (lactate) were measured using an optical plate reader (BMG Labtech, Allmendgrün, Ortenberg). ATP and lactate concentrations were extrapolated from standard curves. Each experimental sample was measured in triplicate and the experiment repeated three times.

### RT^2^ PCR array analysis

Total RNA was extracted from HSR-GBM1 cells and hNSCs using the RNeasy Kit (Qiagen), according to the manufacturer's protocol. cDNA was synthesized using the RT^2^ First Strand Kit (SABiosciences, Frederick, MD, USA), as previously described.^48^ Undifferentiated and day 25 and 50 mtDNA depleted HSR-GBM1 cells were analyzed using the Neurogenesis and Neural Stem Cell RT^2^ Profiler PCR Array (SABiosciences).

Real-time PCR array reactions were performed in triplicate using 384-well (4 × 96) optical reaction plates (SABiosciences). A PCR master mix was prepared per sample containing 102 *μ*l cDNA, 550 *μ*l RT^2^ SYBR Green/ROX Mix (SABiosciences) and 448 *μ*l ddH_2_O. In all, 10 *μ*l PCR reaction mixtures were prepared using a CAS-1200 Robotic Liquid Handling System (Corbett Robotics, Queensland, Australia). Real-time reactions were conducted on an ABI PRISM 7900 HT Fast Real-Time PCR System (Applied Biosystems, Carlsbad, CA, USA) and consisted of an initial denaturation step of 95 °C for 10 min; and 40 cycles of denaturation at 95 °C for 15 s; and an annealing/extension phase at 60 °C for 1 min. Gene expression data were generated in the form of cycle threshold (Ct) values. Relative gene expression was calculated by the ΔΔCt method and normalized against the average Ct values of five housekeeping genes (*glucuronidase beta* (*GUSB*), *hypoxanthine phosphoribosyltransferase 1* (*HPRT1), heat shock protein 90 kDa alpha* (*cytosolic*)*, class B member 1* (*HSP90AB1*), *glyceraldehyde-3-phosphate dehydrogenase* (*GAPDH*) and *beta cytoskeletal actin* (*β-ACTIN*)), according to the manufacturer's protocol. Analyses were performed using the Web-Based PCR Array Data Analysis software (SABiosciences).

### Xenograft models

Mouse experiments were approved by the Animal Ethics Committee, Monash University, Approval Number: MMCA/2011/76. HSR-GBM1 tumor cells (5 × 10^5^) in 100 *μ*l of growth medium were inoculated subcutaneously into both flanks of 5- to 6-week-old, female BALB/c nude mice (Animal Research Centre, Perth, Australia). Tumor volume was determined by: (length × width^2^)/2, where the length was the longest axis and width was measured at right angles to the length. Data are expressed as mean tumor volume±S.E.M. in cubic millimeters.

### Immunohistochemistry and immunocytochemistry

For immunocytochemical analysis, cells were fixed with 4% (weight per volume) paraformaldehyde for 15 min, washed and permeablized with 1% Triton X-100 (Sigma) for 30 min at room temperature. To block nonspecific binding sites, cells were incubated with 4% (volume per volume, v/v) normal goat serum in phosphate-buffered saline (Gibco) with 0.01% (v/v) Tween (Sigma) overnight at 4 °C. Cells were washed and incubated with primary antibodies for NESTIN (1 : 700; Millipore) and GFAP (1 : 300; Millipore) overnight at 4 °C and secondary antibodies (1 : 500; Alexafluor, Invitrogen) for 1 h in darkness. Cells were mounted with prolong antifade 4',6-diamidino-2-phenylindole (Invitrogen) in the dark for 24 h before visualization. Fluorescent microscopy was performed using the Deltavision Deconvolution (Applied Precision Inc., Seattle, WA, USA) workstation mounted on an IX71 Olympus microscope (Olympus, Tokyo, Japan).

Proliferating nuclei were identified using a mouse monoclonal anti-proliferating cell nuclear antigen antibody (1 : 1000; Cell Signaling Technology, Danvers, MA, USA). Formalin-fixed paraffin-embedded sections (5 *μ*m) were dewaxed, rehydrated and microwaved in citrate buffer for antigen retrieval. Once cooled, the sections were incubated with 3% hydrogen peroxide in methanol for 15 min to quench endogenous peroxidase. All sections were then incubated with the DAKO protein blocking solution (Dako Australia, Kingsgrove, Australia) to prevent nonspecific binding. The negative control was performed by deleting the primary antibody. The antibodies were incubated for 1 h at room temperature. The proliferating cell nuclear antigen was visualized with Link Label-Horseradish Peroxidase system by DAKO, according to the manufacturer's protocol (Dako Australia), followed by the chromogen Vector Red for 15 min (Vector Laboratories; Burlingame, CA, USA).

Image analysis was performed using a Leica (Wetzlar, Germany) inverted bright field microscope. Sections were scanned at low magnification to identify areas of high proliferation (hot spots). Images were then captured at × 40 optical lens. Positive nuclei were counted from four fields of view. Using the Image J analysis software (National Institute of Health, Bethesda, MD, USA), positive nuclei were counted using the cell counter plug in analysis tool and the results were presented as mean values±S.E.M.

### Western blotting

Cell pellets were resuspended in lysis buffer (25 mM Tris-chloride pH 7.6, 150 mM NaCl, 1% (v/v) Triton-X-100, 0.1% (v/v) sodium dodecyl sulfate, 1 mM phenylmethanesulfonyl fluoride, 1 x complete protease inhibitor (Roche, Mannheim, Germany)) and incubated on ice for 30 min. Following centrifugation at 4 °C for 30 min, the supernatant was transferred to a new tube and the protein concentration estimated. Proteins were precipitated with 12% (v/v) trichloroacetic acid for 30 min on ice, pelleted at 16 000 *g*, rinsed twice with ice cold acetone before being resuspended in Laemmli Buffer (50 mM Tris-chloride pH 6.8, 100 mM dithiothreitol, 2% (v/v) sodium dodecyl sulfate, 10% (v/v) glycerol). Samples were separated using the Tris-tricine gel system, as described previously.^49^ The gel was then transferred to polyvinylidene difluoride membrane (Millipore) using the semi-dry method as outlined previously before immunodecoration with the indicated antibodies,^50^ OPA1 (BD Biosciences, Franklin Lakes, NJ, USA) and LC3B (Cell Signaling Technology).

### Statistical analysis

Statistical significance for the RT^2^ PCR arrays was determined using the Web-Based PCR Array Data Analysis software (SABiosciences), which used a two-tailed Student's *t*-test. For real-time PCR and tumor assays, statistically significant differences were determined using one-way ANOVA followed by Bonferroni *post hoc* test using GraphPad v5.0c (GraphPad Software, Inc., San Deigo, CA, USA). Statistical significance is expressed as *P<0.05, **P<0.01 and ***P<0.001.

## Abbreviations

mtDNA: mitochondrial DNA/mitochondrial genome
GBM: glioblastoma multiforme
hNSCs: human neural stem cells
ATP: adenosine triphosphate
ETC: electron transfer chain
OXPHOS: oxidative phosphorylation
TFAM: mitochondrial transcription factor A
POLGA: polymerase *γ* A
POLGB: polymerase *γ* B
MTSSB: mtDNA single-stranded-binding protein
GFAP: glial fibrillary acidic protein
O_2_: oxygen
ddC: 2'-3'-dideoxycytidine
*FGF13*: fibroblast growth factor 13
*GDNF*: glial-derived neurotrophic factor
*SEMA4D*: semaphorin-4D
*VEGFA*: vascular endothelial growth factor A
*SHH*: sonic hedgehog
*ACHE*: acetylcholinesterase
*DRD2*: dopamine receptor D2
*NPTX*: neuronal pentraxin 1
*OCT4*: POU class 5 homeobox 1
*NANOG*: Nanog homeobox
*SOX2*: sex determining region Y-box 2
*c-MYC*: V-Myc myelocytomatosis viral oncogene homolog (avian)
*hTERT*: human telomerase reverse transcriptase
*CD133*: prominin 1
Balb/c: Bagg albino
OPA1: optic atrophy 1
LC3B: microtubule-associated protein 1 light chain 3 beta
Basal: initial resting measurements
Non-phos: non-phosphorylating respiration
Uncoupled: maximal uncoupled ETC respiratory capacity
FCCP: carbonyl cyanide p-(trifluoromethoxy)-(phenylhydrazone)
Ct: cycle threshold
v/v: volume per volume
