# Supplementary figures and images for: The regulation of mitochondrial DNA copy number in glioblastoma cells
Source: Cell Death Differ. 2013 Aug 30;20(12):1644–53. doi: 10.1038/cdd.2013.115 (PMC3824586; doi:10.1038/cdd.2013.115)

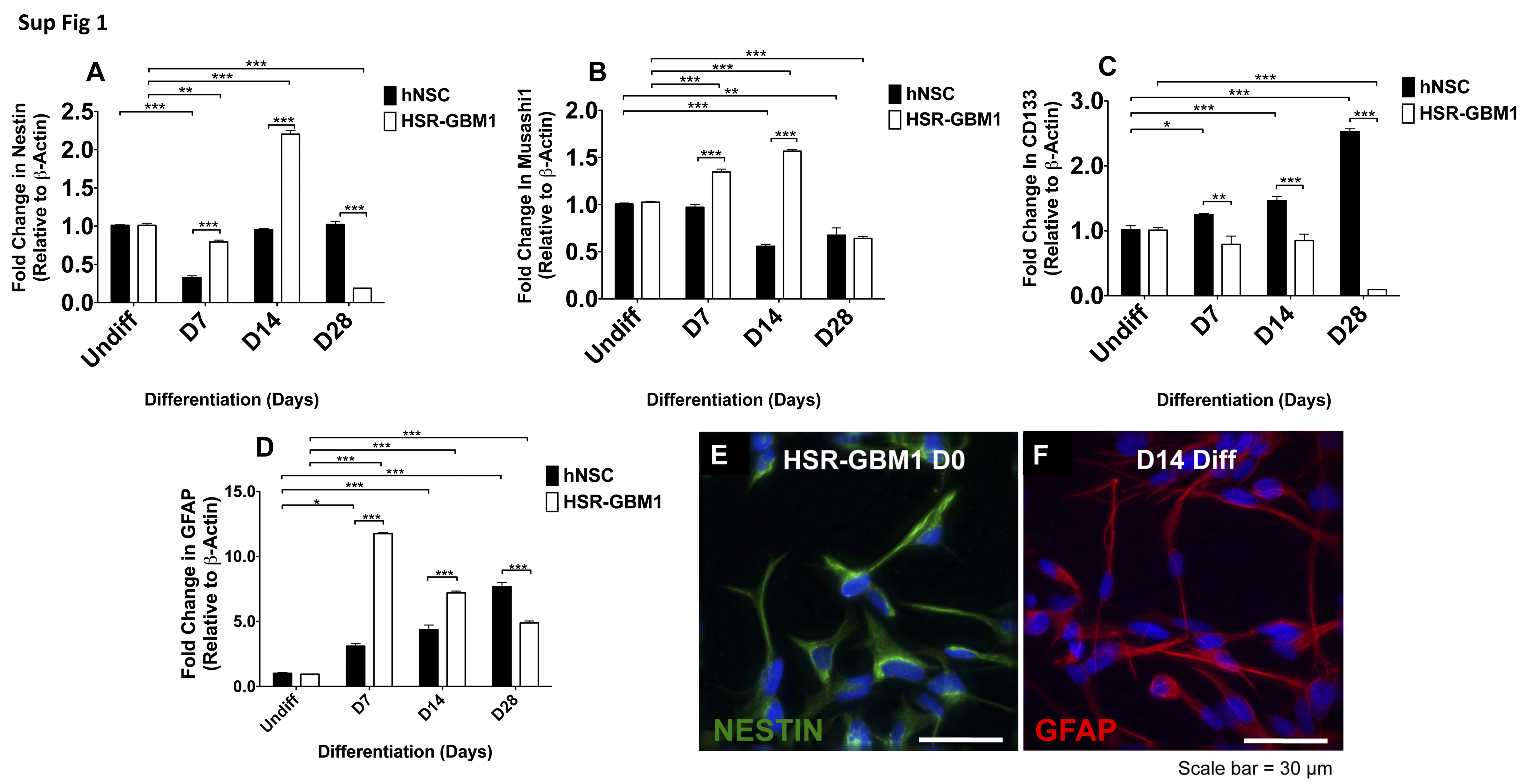

Supplement: Supplementary Figure S1 [file cdd2013115x1.tif]

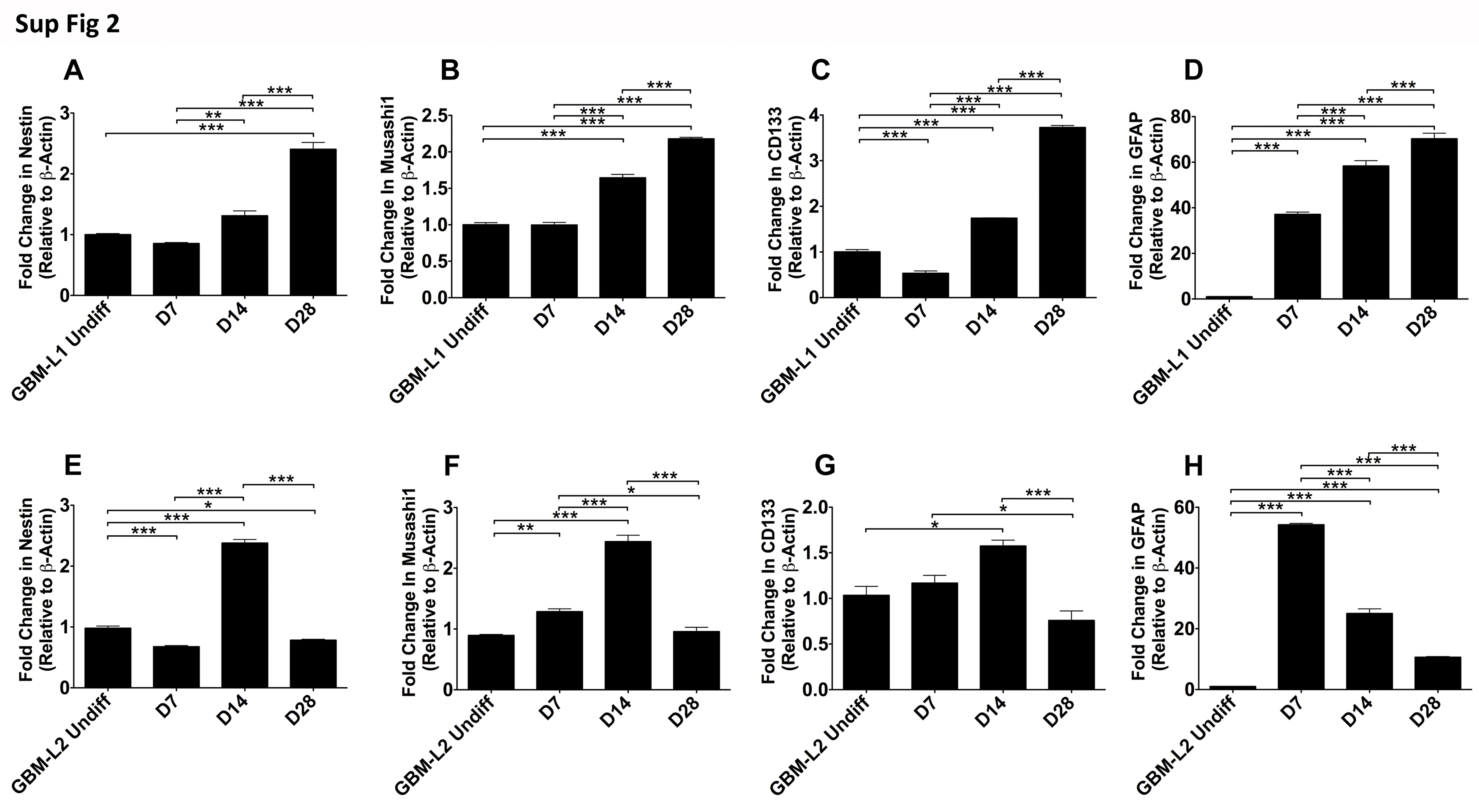

Supplement: Supplementary Figure S2 [file cdd2013115x2.tif]

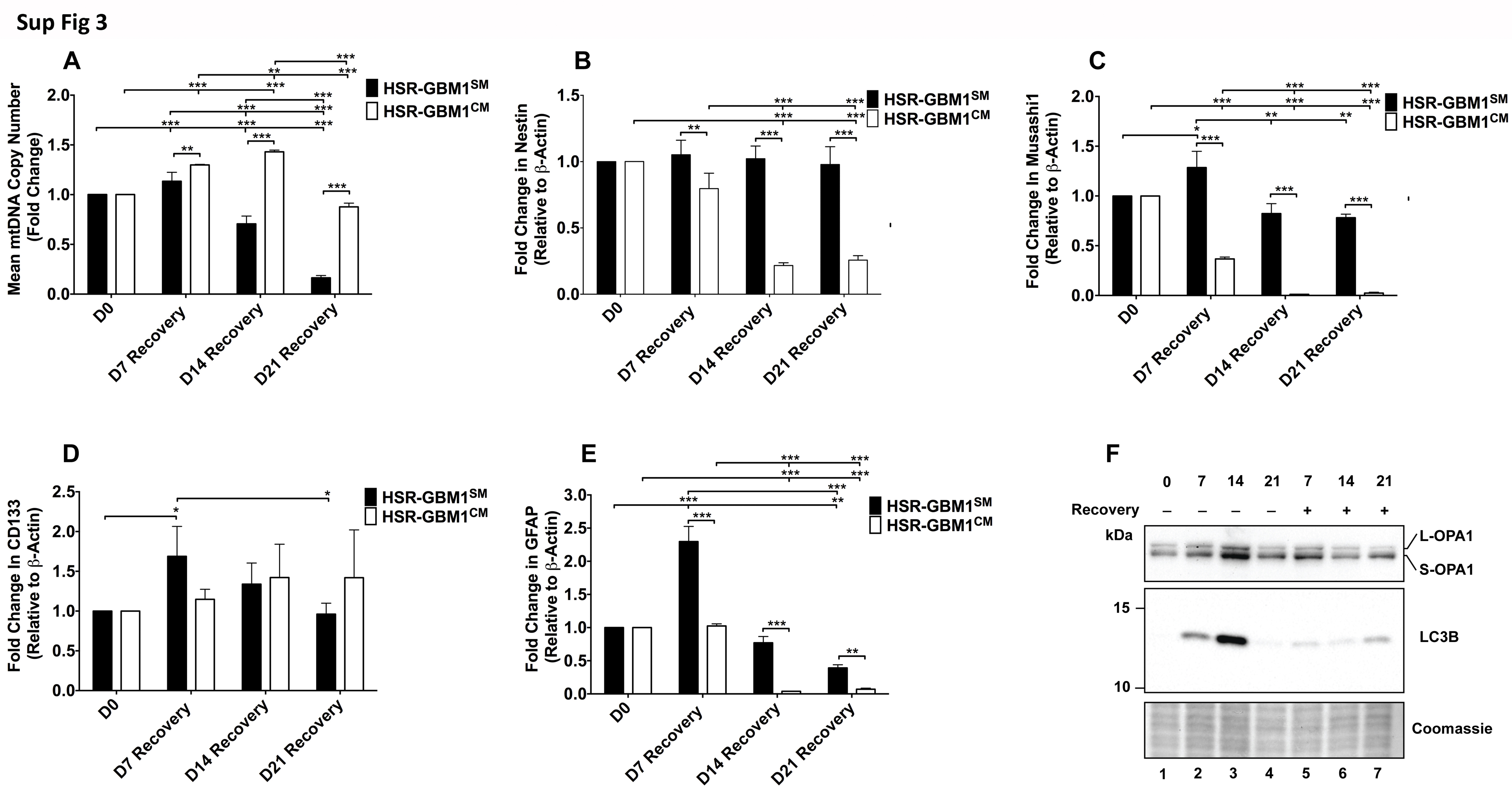

Supplement: Supplementary Figure S3 [file cdd2013115x3.tif]

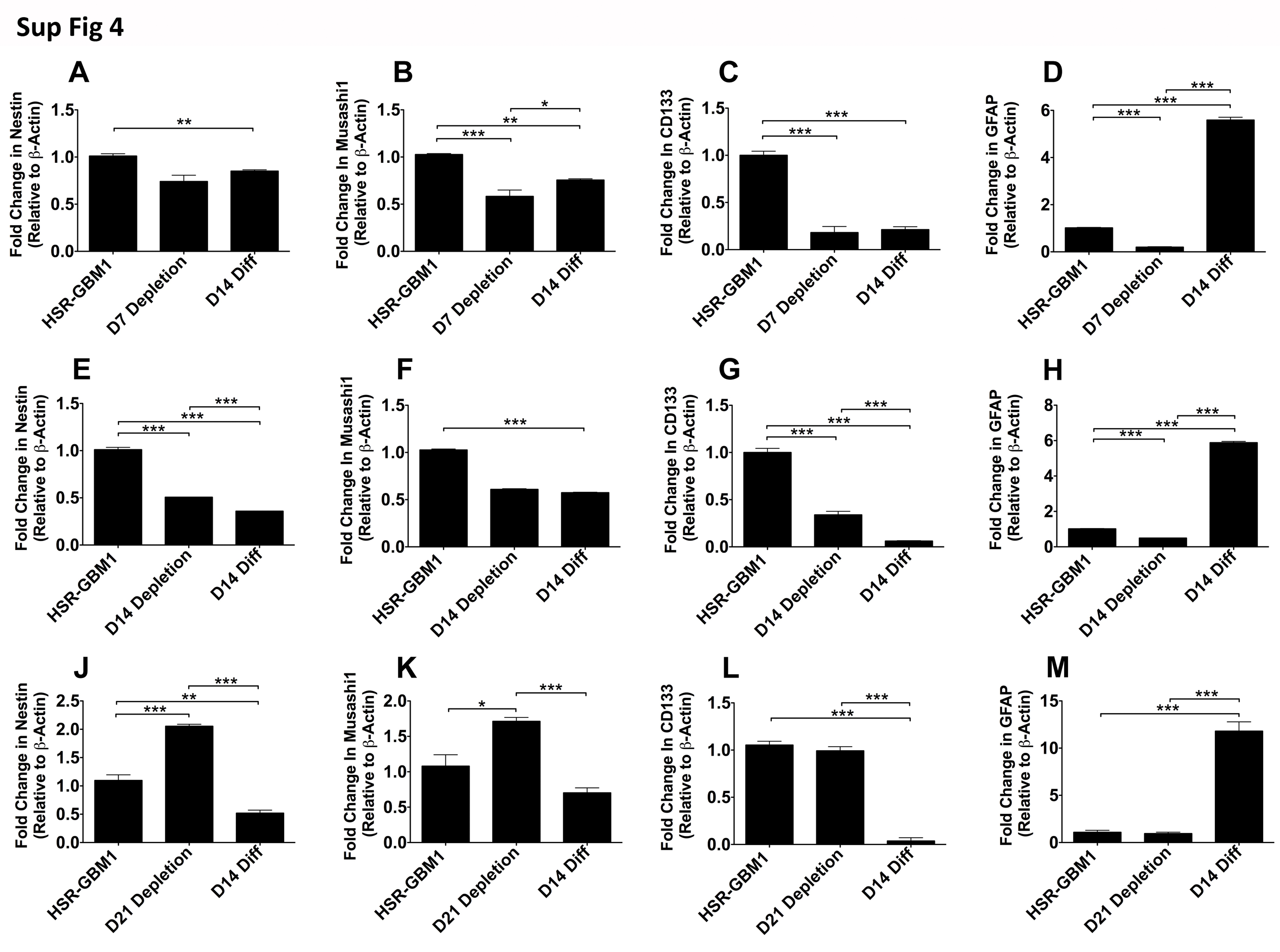

Supplement: Supplementary Figure S4 [file cdd2013115x4.tif]
