# Supplementary material for: The regulation of mitochondrial DNA copy number in glioblastoma cells
Source: Cell Death Differ. 2013 Aug 30;20(12):1644–53. doi: 10.1038/cdd.2013.115 (PMC3824586; doi:10.1038/cdd.2013.115)
Supplement: Supplementary Tables [file cdd2013115x5.doc]

|  | **O_2_ Consumption**  **(pmol/per sec/10^6^ cells)** | | | **ETC Reserve Capacity** | **ATP Content** | **Lactate Production** |
| --- | --- | --- | --- | --- | --- | --- |
|  | **Basal** | **Non-Phosphorylating** | **Uncoupled** | **Uncoupled:**  **Basal** | **(nM/cell)** | **(mM/10^5^ cells)** |
| **HSR-GBM1**  **Undifferentiated** | 21.4 ± 0.9 | 9.9 ± 0.5 | 23.0 ± 1.0 | 1.1 ± 0.05 | 0.14 ± 0.01 ^a*** c*^ | 2.2 ± 0.2 |
| **HSR-GBM1**  **Differentiated** | 24.7 ± 2.8 | 11.6 ± 1.0 | 30.8 ± 3.9 | 1.2 ± 0.02 | 0.23 ± 0.03 | 0.2 ± 0.03 ^a***^ |
| **hNSC**  **Undifferentiated** | 8.9 ± 1.0 ^c***^ | 4.4 ± 0.6 | 6.9 ± 0.8 | 0.9 ± 0.02 ^b*** c*^ | 0.1 ± 0.002 ^b***^ | 0.7 ± 0.03 ^c***^ |
| **hNSC**  **Differentiated** | 21.9 ± 1.6 ^b***^ | 7.1 ± 1.0 | 38.9 ± 3.4 | 1.9 ± 0.02 ^d***^ | 0.4 ± 0.01  ^d***^ | 0.5 ± 0.02 |

**Supplementary Table I.**

^a^ Significance between GBM Undifferentiated and GBM Differentiated

^b^ Significance between hNSC Undifferentiated and hNSC Differentiated

^c^ Significance between GBM Undifferentiated and hNSC Undifferentiated

^d^ Significance between GBM Differentiated and hNSC Differentiated

* Indicates p<0.05, ** p<0.01 and *** p<0.001

**Supplementary Table II.**

| **Genes** | **Day 25 Depletion** | **Day 50 Depletion** | **Gene Function** |
| --- | --- | --- | --- |
| *ACHE* | 4.877 | 2.9752 | Cell Proliferation, Cell Adhesion, Synaptogenesis |
| *ALK* | 1.8307 | 2.2425 | Negative regulator of proliferation |
| *APOE* | -1.3745 | -3.1814 | Synaptogenesis |
| *ASCL1* | -1.4529 | -4.8572 | Regulator of differentiation and transcription |
| *CDK5RAP3* | 1.6607 | 2.0013 | Cell proliferation, Neuronal differentiation |
| *DLG4* | 3.0939 | 1.5442 | Synaptogenesis |
| *DLL1* | -1.7325 | -3.2369 | Cell fate, Cell adhesion, Notch signalling pathway |
| *DRD2* | 8.7294 | 3.448 | Synaptogenesis |
| *EFNB1* | 1.0598 | -2.1937 | Cell Adhesion |
| *ERBB* | 3.0099 | 1.9495 | Cell Adhesion |
| *FGF13* | 2.6468 | 2.9754 | Growth Factor |
| *GDNF* | 4.9007 | 4.3017 | Growth Factor, Anti-apoptosis |
| *HEY1* | 4.1459 | 5.0557 | Transcription Factor |
| *HEY2* | 1.0118 | -2.2584 | Transcription Factor |
| *NPTX* | 7.3376 | 3.7001 | Synaptogenesis |
| *NRCAM* | 2.4282 | 2.6759 | Cell adhesion, Cell differentiation |
| *NRP1* | 2.0396 | 1.2941 | Cell Adhesion |
| *NRP2* | 3.1056 | 1.569 | Cell Adhesion |
| *NTN1* | 2.0472 | 1.392 | Anti-apoptosis |
| *PARD6B* | 2.1265 | 1.4166 | Cell cycle |
| *S100A6* | -1.314 | 2.5712 | Cell proliferation, Cell Cycle, Cell differentiation |
| *SEMA4D* | 5.1751 | 5.0328 | Cell adhesion, Anti-apoptosis |
| *SOX8* | -1.2876 | -2.3618 | Development, Apoptosis, Oligodendrocyte differentiation |
| *SHH* | Not Detectable | 3.8904 | Development |
| *TNR* | 2.0176 | 1.6121 | Cell Adhesion |
| *VEGFA* | 2.2613 | 1.6458 | Anti-apoptosis, Growth factor, Cell Proliferation |

**Supplementary Table III.**

| **Gene** | **Forward Primer** | **Reverse Primer** | **Product Size (bp)** |
| --- | --- | --- | --- |
| *β-ACTIN* | CAA AAC CTA ACT TGC GCA GA | TTT TAG GAT GGC AAG GGA CT | 261 |
| *β-GLOBIN* | caa ctt cat cca cgt tca cc | gaa gag cca agg aca ggt ac | 268 |
| *C-MYC* | ACA ACA CCC GAG CAA GGA CGC | ACG GCT GCA CCG AGT CGT AGT | 180 |
| *CD133* | GCA TTG GCA TCT TCT ATG GTT | CGCCTTGTCCTTGGTAGTGT | 190 |
| *GFAP* | GAA GCT CCA GGA TGA AAC CA | ACC TCC TCC TCG TGG ATC TT | 165 |
| *hTERT* | CAC CAA GAA GTT CAT CTC C | CAA GTG CTG TCT GAT TCC | 260 |
| *mtDNA* | cga aag gac aag aga aat aag g | ctg taa agt ttt aag ttt tat gcg | 152 |
| *MUSASHI1* | AGA AAG CTC AGC CAA AGG AG | GAA TTC GGG GAA CTG GTA GG | 194 |
| *NANOG* | TTA ATA ACC TTG GCT GCC GT | GCA GCA AAT ACG AGA CCT CT | 298 |
| *NESTIN* | AAA CCA GAG CCA TGA GAC AC | TGG CCT ACA GCC TCT TTT TC | 156 |
| *OCT4* | TCA CCC TGG GGG TTC TAT TT | CTG GTT CGC TTT CTC TTT CG | 202 |
| *SOX2* | GGA GCT TTG CAG GAA GTT TG | GCA AGA AGC CTC TCC TTG AA | 191 |
| *MTSSB* | GAA GCC ATG TTT CGA AGA CCT G | CTG ATA TTC TGT GCC ATG TTG TC | 271 |
| *POLGA* | CAC ACC TAA ACT CAT GGC AC | GTC CAC GTC GTT GTA AGG TC | 436 |
| *POLGB* | GTT TGC CAT GAG TCC ATC TAA C | CTC TGT CAG CTG GAA AGA ATC | 280 |
| *TFAM* | ATT GGG GTC GGG TCA CTG CCT CA | TAC CTG CCA CTC CGC CCT ATA AGC | 361 |
| *TWINKLE* | GCA CAA GTC CAT CGT ATC TTT C | CAT ACT CAC TGA TGA ATG TCG TC | 197 |
