# Supplementary material for: The regulation of mitochondrial DNA copy number in glioblastoma cells
Source: Cell Death Differ. 2013 Aug 30;20(12):1644–53. doi: 10.1038/cdd.2013.115 (PMC3824586; doi:10.1038/cdd.2013.115)
Supplement: Supplementary Legend [file cdd2013115x6.doc]

**Supplemental Data**

**Supplementary Figure Legends**

**Figure S1. Gene expression analysis of neural stem cell makers in differentiating hNSCs and HSR-GBM1 cells.** Fold change in expression relative to undifferentiated cells, weighted to *β-ACTIN*, for *NESTIN* (A), *MUSASHI1* (B), *CD133* (C) and *GFAP* (D). Immunocytochemical labeling of Nestin (green; E) and GFAP (red; F) in undifferentiated and differentiated HSR-GBM1 cells, respectively. Nuclei are labeled with DAPI (blue). Columns represent mean values ± SEM. * indicates p<0.05, ** p<0.01 and *** p<0.001. Scale bar = 30 μm.

**Figure S2. Gene expression analysis of neural stem cell makers in differentiating GBM-L1 (A-D) and GBM-L2 (E-H) cells.** Fold change in expression relative to undifferentiated cells, weighted to *β-ACTIN*, for *NESTIN* (A & E), *MUSASHI1* (B & F), *CD133* (C & G) and *GFAP* (D & H). Columns represent mean values ± SEM. * indicates p<0.05, ** p<0.01 and *** p<0.001.

**Figure S3.** **Analysis of depleted and recovering HSR-GBM1 cells.** Mean (± SEM) mtDNA copy number following 14 days of recovery of undifferentiated HSR-GBM1 cells depleted for up to 21 days in standard media (SM) and non-depleted HSR-GBM1 cell conditioned media (CM) (A). Gene expression analysis of recovering HSR-GBM1 cells following 7 to 21 days of depletion weighted against *β-ACTIN* for *NESTIN* (B), *MUSASHI1* (C), *CD133* (D) and *GFAP* (E) relative to non-depleted HSR-GBM1 cells. OPA1 processing and LC3B expression in non-depleted, depleted and recovering HSR-GBM1 cells (F). OPA1 is present at the mitochondrial inner membrane as a complex of both long (L) and short (S) forms and a balance between L- and S-OPA1 is essential for maintaining wild type mitochondrial morphology. In response to mitochondrial stress, L-OPA1 is processed to S-OPA1 preventing mitochondrial fusion, resulting in a fragmented mitochondrial network. Bars represent mean values ± SEM. * indicates p<0.05, ** p<0.01 and *** p<0.001.

**Figure S4. Gene expression analysis of neural stem cell and lineage specific makers in differentiating mtDNA depleted HSR-GBM1 cells.** HSR-GBM1 cells were depleted for 7 days (A-D), 14 days (E-H) and 21 days (J-M) and differentiated for 14 days. Gene expression was relative to non-depleted cells and weighted to *β-ACTIN* for *NESTIN* (A, E, J), *MUSASHI1* (B, F, K), *CD133* (C, G, L) and *GFAP* (D, H, M). The columns represent mean values ± SEM. * indicates p<0.05, ** p<0.01 and *** p<0.001.

**Table SI.** O_2_ consumption rates, cellular ATP content and lactate production for undifferentiated and differentiated hNSCs and HSR-GBM1 cells. Statistical significance as indicated.

**Table SII.** **Summary of gene expression analysis from the Neurogenesis and Neural Stem Cell PCR Array.** Fold changes in gene expression of undifferentiated HSR-GBM1 cells and undifferentiated HSR-GBM1 cells depleted for 25 and 50 days. Statistically significant (p<0.05) increases in expression are shown in red and decreases in expression in blue.

**Table SIII. RT-PCR and real-time PCR primer sequences and product sizes.**
